# Mob Law and Its Remedy

**Published:** 1897-09

**Authors:** 


### EDITORIAL.

The office of the Business Manager of THE JOURNAL is 311 and 312 Fitten Building.

The editors are not responsible for views expressed by contributors.

Articles for publication should reach this office not later than the 15th inst.

Address all communications, and make all remittances payable to THE ATLANTA MEDICAL AND SURGICAL JOURNAL, Atlanta, Ga.

Reprints of articles will be furnished, when desired, at a small cost. Requests for the same should always be made *on the manuscript*.

### MOB LAW AND ITS REMEDY.

Within the last few months a wave of lawlessness has passed over our country, generally taking the form of mob vengeance on prisoners. In some cases the violence has been inflicted after the victims have been tried and received the extreme sentence of the law, in others they have been taken red-handed or have confessed their guilt, and in one case no specific charge was brought, but death was meted out "on general principles." Such a state of affairs is lamentable from many points of view: from that of abstract morality, because an execution of this character is murder, cold-blooded and premeditated; from that of civilization, as evincing a disregard for law and order and indicating an incapability for self-government; and from a pecuniary, as it discourages the coming of immigrants and capital among us.

Such being the case, it is of great importance for thoughtful men throughout the South to look into the causes of this lawlessness with a view to removing it. Many causes are alleged and it is impossible to consider all of them, but many writers agree that our code of criminal procedure is at fault, and that the people become exasperated by the many loopholes through which the prisoners escape the penalties of their crimes, even in cases in which there is no reasonable doubt of their guilt. The opportunities for indefinite delay and the trivial reasons assigned for continuance or retrial with the accompanying heavy bill of costs for the commonwealth, are among the reasons given for the growing distrust of the courts. The writer remembers a case in which he was a witness that illustrates this point. The prisoner had been indicted by the grand jury, and the indictment duly drawn by the proper officer. At the time of the trial a copy of this document was read by the clerk and it was found that in the copy there was an error. The bill read that the prisoner struck his victim with a knife, thereby inflicting a fatal wound and that "of that fatal wound he did die," etc. The stenographer, in copying this, omitted the word "wound," leaving it to read that "of that fatal, he did die," etc., and on account of that error in a copy, the witnesses were taken from their own business affairs a second time and the State put to additional expense. Why a new copy could not have been made at once is not apparent to an ordinary mortal, especially as the law proudly asserts "*De minimis non curat lex.*"

To whom are we to look for relief? There is but one reply—to the lawyers. Most of our laws are made by them and for them, and most of the delays of which the people complain are granted for their convenience. When a contractor fails to comply with the terms of his contract, he cannot claim that he was so much engaged on other work that he was unable to complete a certain undertaking in the time agreed. He would be told to confine his contracts to his ability to fulfill them; but if a lawyer claims that he is not prepared for trial because of the time put on other cases, the plea is accepted and the case postponed, no matter how serious the inconvenience to which all of the interested parties may be put. It is generally difficult for a busy man to leave his work, particularly if he be a professional man, but bench and bar have no consideration for those without their own charmed circle, and the very men who would be indignant if a physician plead fatigue or overwork as an excuse for tardiness in answering a call to relieve one of them of pain or sickness, would think them excellent reasons for postponing a trial, regardless of the inconvenience that may thereby be caused a large number of persons.

If these objections be raised, they will say that their business is to practise the law as it is, not to rectify it, or they will say that the law is perfect, that every tort has a remedy at law. The reply reminds one of the statement of the Duke of Wellington before the Reform Bill of 1832, that parliamentary representation in Great Britain was perfect, and the fate of the duke should be a lesson to the confident jurists. The frequent violations of the majesty of the law show that something is wrong, and we have a right to expect from the professed students of jurisprudence some indication of the way to remedy the defect.

Up to the beginning of this century, epidemics, more destructive than wars, swept over the world from time to time, and raged unchecked till all of the material on which the disease lived was destroyed. Such is no longer the case. Smallpox has practically been exterminated, diphtheria has lost its chief terrors; four years ago typhus fever appeared in New York, but was confined to a single house, and only last winter the bubonic plague started on its disastrous course but was stopped with a promptness that was truly wonderful. Crime, however, rages unhindered, and often after the crime of the individual comes the crime of the many, and those who profess to be to social ills what physicians are to bodily ones, stand idle and inert. It is true that in its widest sense, the prevention of crime would diminish litigation, but the prevention of disease has diminished the work of physicians, and the bar will hardly grant to the medical profession a monopoly of altruism and humanity.

Will not our bench and bar rise to a true patriotism and see how far our present criminal methods are responsible for mob violence? If the charge so often made be true, they should take the necessary steps to correct the errors and so rid our fair land of this terrible blot. *

---

Here is a new feature in medical college catalogues, though in execrable taste. A school in the far West informs the reader that "the members of the faculty are all mature and well-known physicians, etc. Professor —— is one of the best known microscopists and bacteriologists in the United States; . . . Professor —— is a well-known surgeon and expert on railway surgery; . . . Professor —— is the well-known ophthalmologist of ——; . . . Professor —— has spent many years a laborer in the field of medical education. He is author of the section on Diseases of the Lungs in the —— Practice of Medicine, and also of the Prize Essay of the —— Medical Society, on Mineral Springs and Health Resorts of ——. Professor —— is known to almost every medical student in the English-speaking world by the —— Series of Quiz-Compends which he designed and two of which he wrote, etc. In 1887 was published his Handbook of ——, a volume of 900 pages. . . . In 1891 Professor —— received the diploma of Member of the Royal College of Physicians of London, by examination, upon a purely American medical education." Etc., etc., with the balance of the men who compose this distinguished faculty. Which shows that the spirit of the code is becoming a back number out in the hustling community of the Golden Gate.

